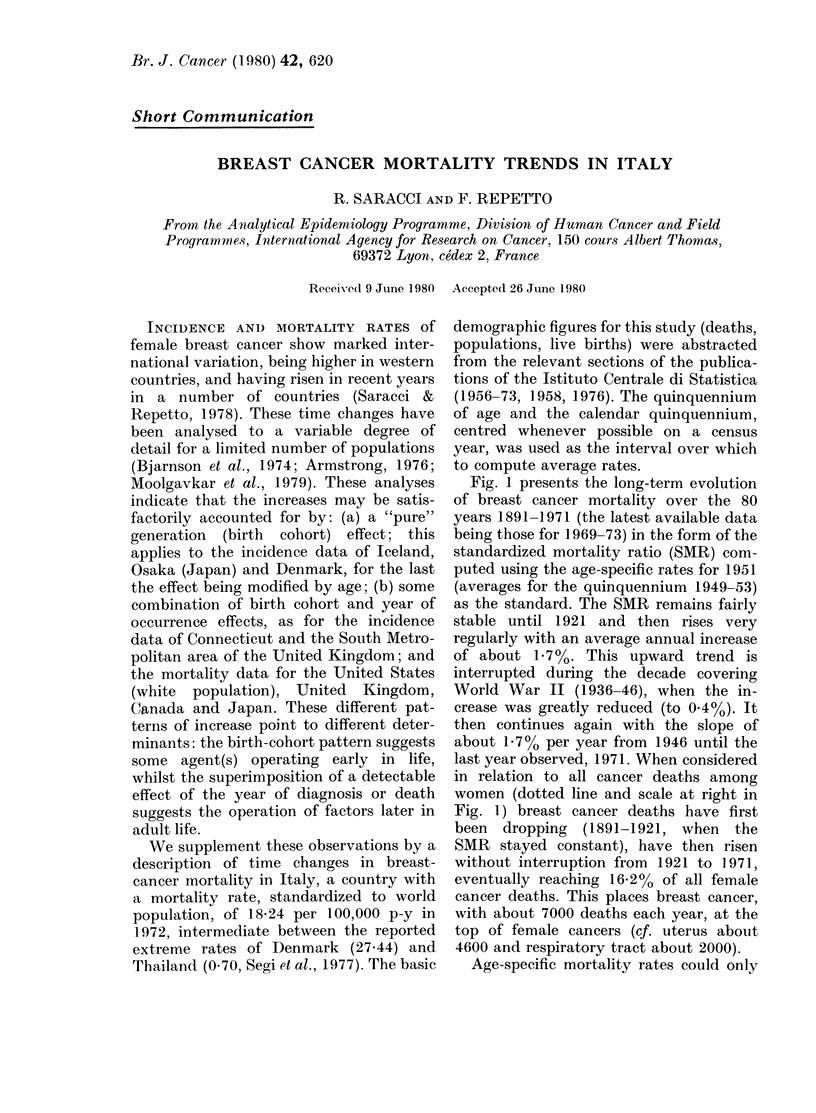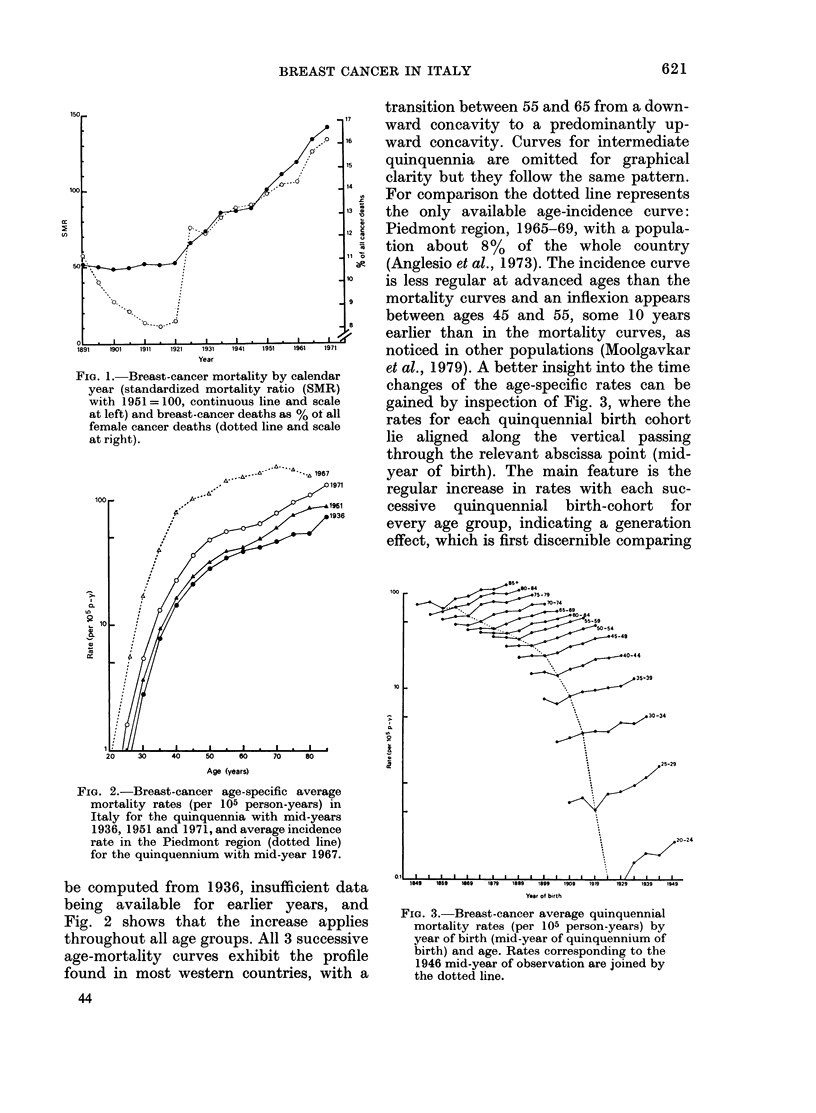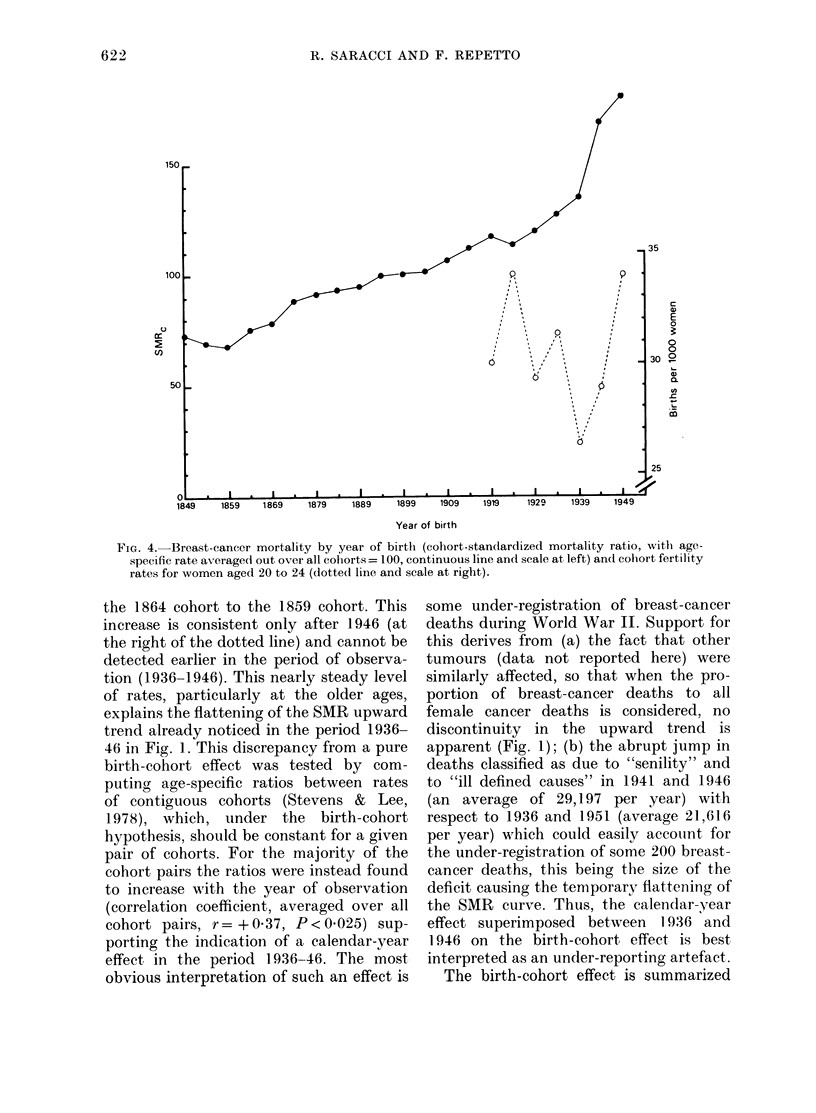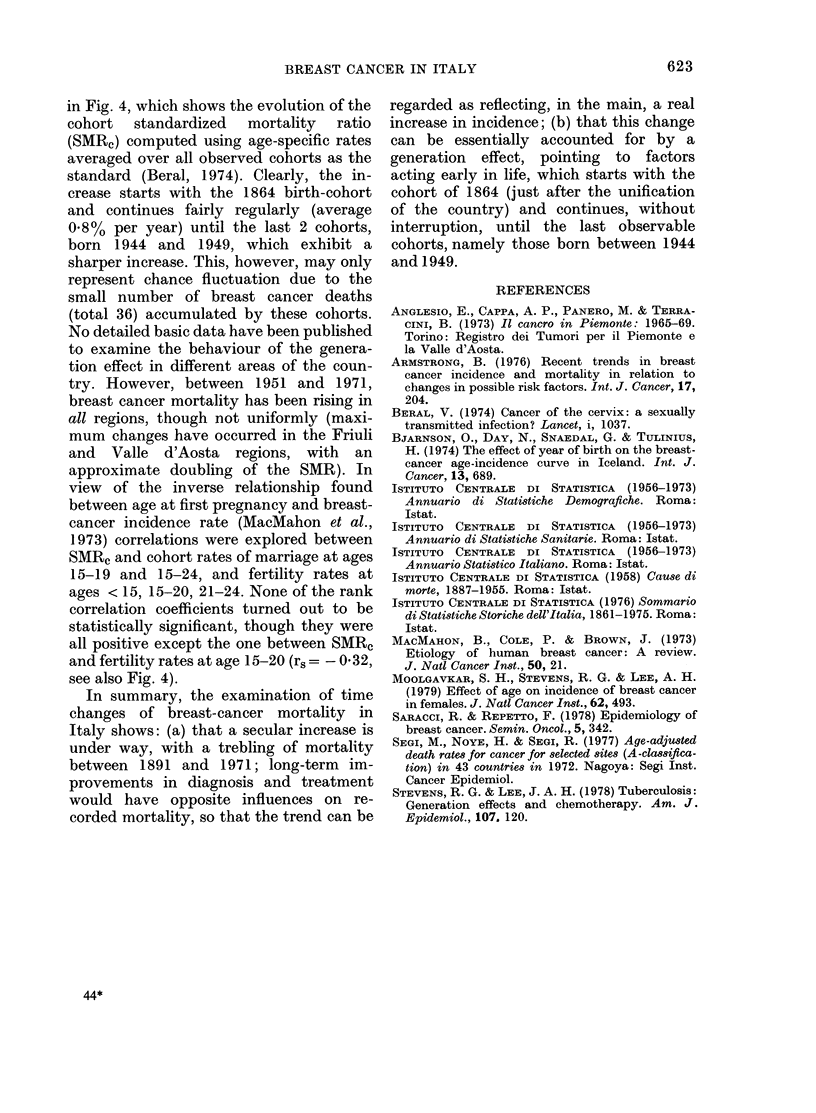# Breast cancer mortality trends in Italy.

**DOI:** 10.1038/bjc.1980.288

**Published:** 1980-10

**Authors:** R. Saracci, F. Repetto


*Br. J. Cancer* (1980) **42**, 620

***Short Communication***

### BREAST CANCER MORTALITY TRENDS IN ITALY

R. SARACCI AND F. REPETTO

*From the Analytical Epidemiology Programme, Division of Human Cancer and Field Programmes, International Agency for Research on Cancer,* 150 *cours Albert Thomas,* 69372 *Lyon, cédex* 2, *France*

Received 9 June 1980 Accepted 26 June 1980

INCIDENCE AND MORTALITY RATES of female breast cancer show marked international variation, being higher in western countries, and having risen in recent years in a number of countries (Saracci & Repetto, 1978). These time changes have been analysed to a variable degree of detail for a limited number of populations (Bjarnson *et al.*, 1974; Armstrong, 1976; Moolgavkar *et al.*, 1979). These analyses indicate that the increases may be satisfactorily accounted for by: (a) a "pure" generation (birth cohort) effect; this applies to the incidence data of Iceland, Osaka (Japan) and Denmark, for the last the effect being modified by age; (b) some combination of birth cohort and year of occurrence effects, as for the incidence data of Connecticut and the South Metropolitan area of the United Kingdom; and the mortality data for the United States (white population), United Kingdom, Canada and Japan. These different patterns of increase point to different determinants: the birth-cohort pattern suggests some agent(s) operating early in life, whilst the superimposition of a detectable effect of the year of diagnosis or death suggests the operation of factors later in adult life.

We supplement these observations by a description of time changes in breast-cancer mortality in Italy, a country with a mortality rate, standardized to world population, of 18·24 per 100,000 p-y in 1972, intermediate between the reported extreme rates of Denmark (27·44) and Thailand (0·70, Segi *et al.*, 1977). The basic demographic figures for this study (deaths, populations, live births) were abstracted from the relevant sections of the publications of the Istituto Centrale di Statistica (1956–73, 1958, 1976). The quinquennium of age and the calendar quinquennium, centred whenever possible on a census year, was used as the interval over which to compute average rates.

Fig. 1 presents the long-term evolution of breast cancer mortality over the 80 years 1891–1971 (the latest available data being those for 1969–73) in the form of the standardized mortality ratio (SMR) computed using the age-specific rates for 1951 (averages for the quinquennium 1949–53) as the standard. The SMR remains fairly stable until 1921 and then rises very regularly with an average annual increase of about 1·7%. This upward trend is interrupted during the decade covering World War II (1936–46), when the increase was greatly reduced (to 0·4%). It then continues again with the slope of about 1·7% per year from 1946 until the last year observed, 1971. When considered in relation to all cancer deaths among women (dotted line and scale at right in Fig. 1) breast cancer deaths have first been dropping (1891–1921, when the SMR stayed constant), have then risen without interruption from 1921 to 1971, eventually reaching 16·2% of all female cancer deaths. This places breast cancer, with about 7000 deaths each year, at the top of female cancers (*cf.* uterus about 4600 and respiratory tract about 2000).

Age-specific mortality rates could only

FIG. 1.—Breast-cancer mortality by calendar year (standardized mortality ratio (SMR) with 1951 = 100, continuous line and scale at left) and breast-cancer deaths as % of all female cancer deaths (dotted line and scale at right).

FIG. 2.—Breast-cancer age-specific average mortality rates (per $10^5$ person-years) in Italy for the quinquennia with mid-years 1936, 1951 and 1971, and average incidence rate in the Piedmont region (dotted line) for the quinquennium with mid-year 1967.

be computed from 1936, insufficient data being available for earlier years, and Fig. 2 shows that the increase applies throughout all age groups. All 3 successive age-mortality curves exhibit the profile found in most western countries, with a transition between 55 and 65 from a downward concavity to a predominantly upward concavity. Curves for intermediate quinquennia are omitted for graphical clarity but they follow the same pattern. For comparison the dotted line represents the only available age-incidence curve: Piedmont region, 1965–69, with a population about 8% of the whole country (Anglesio *et al.*, 1973). The incidence curve is less regular at advanced ages than the mortality curves and an inflexion appears between ages 45 and 55, some 10 years earlier than in the mortality curves, as noticed in other populations (Moolgavkar *et al.*, 1979). A better insight into the time changes of the age-specific rates can be gained by inspection of Fig. 3, where the rates for each quinquennial birth cohort lie aligned along the vertical passing through the relevant abscissa point (mid-year of birth). The main feature is the regular increase in rates with each successive quinquennial birth-cohort for every age group, indicating a generation effect, which is first discernible comparing

FIG. 3.—Breast-cancer average quinquennial mortality rates (per $10^5$ person-years) by year of birth (mid-year of quinquennium of birth) and age. Rates corresponding to the 1946 mid-year of observation are joined by the dotted line.

FIG. 4.—Breast-cancer mortality by year of birth (cohort-standardized mortality ratio, with age-specific rate averaged out over all cohorts = 100, continuous line and scale at left) and cohort fertility rates for women aged 20 to 24 (dotted line and scale at right).

the 1864 cohort to the 1859 cohort. This increase is consistent only after 1946 (at the right of the dotted line) and cannot be detected earlier in the period of observation (1936–1946). This nearly steady level of rates, particularly at the older ages, explains the flattening of the SMR upward trend already noticed in the period 1936–46 in Fig. 1. This discrepancy from a pure birth-cohort effect was tested by computing age-specific ratios between rates of contiguous cohorts (Stevens & Lee, 1978), which, under the birth-cohort hypothesis, should be constant for a given pair of cohorts. For the majority of the cohort pairs the ratios were instead found to increase with the year of observation (correlation coefficient, averaged over all cohort pairs, $r = +0{\cdot}37$, $P < 0{\cdot}025$) supporting the indication of a calendar-year effect in the period 1936–46. The most obvious interpretation of such an effect is some under-registration of breast-cancer deaths during World War II. Support for this derives from (a) the fact that other tumours (data not reported here) were similarly affected, so that when the proportion of breast-cancer deaths to all female cancer deaths is considered, no discontinuity in the upward trend is apparent (Fig. 1); (b) the abrupt jump in deaths classified as due to "senility" and to "ill defined causes" in 1941 and 1946 (an average of 29,197 per year) with respect to 1936 and 1951 (average 21,616 per year) which could easily account for the under-registration of some 200 breast-cancer deaths, this being the size of the deficit causing the temporary flattening of the SMR curve. Thus, the calendar-year effect superimposed between 1936 and 1946 on the birth-cohort effect is best interpreted as an under-reporting artefact.

The birth-cohort effect is summarized in Fig. 4, which shows the evolution of the cohort standardized mortality ratio ($SMR_c$) computed using age-specific rates averaged over all observed cohorts as the standard (Beral, 1974). Clearly, the increase starts with the 1864 birth-cohort and continues fairly regularly (average 0·8% per year) until the last 2 cohorts, born 1944 and 1949, which exhibit a sharper increase. This, however, may only represent chance fluctuation due to the small number of breast cancer deaths (total 36) accumulated by these cohorts. No detailed basic data have been published to examine the behaviour of the generation effect in different areas of the country. However, between 1951 and 1971, breast cancer mortality has been rising in *all* regions, though not uniformly (maximum changes have occurred in the Friuli and Valle d'Aosta regions, with an approximate doubling of the SMR). In view of the inverse relationship found between age at first pregnancy and breast-cancer incidence rate (MacMahon *et al.*, 1973) correlations were explored between $SMR_c$ and cohort rates of marriage at ages 15–19 and 15–24, and fertility rates at ages $<15$, 15–20, 21–24. None of the rank correlation coefficients turned out to be statistically significant, though they were all positive except the one between $SMR_c$ and fertility rates at age 15–20 ($r_s = -0{\cdot}32$, see also Fig. 4).

In summary, the examination of time changes of breast-cancer mortality in Italy shows: (a) that a secular increase is under way, with a trebling of mortality between 1891 and 1971; long-term improvements in diagnosis and treatment would have opposite influences on recorded mortality, so that the trend can be regarded as reflecting, in the main, a real increase in incidence; (b) that this change can be essentially accounted for by a generation effect, pointing to factors acting early in life, which starts with the cohort of 1864 (just after the unification of the country) and continues, without interruption, until the last observable cohorts, namely those born between 1944 and 1949.

### REFERENCES

Anglesio, E., Cappa, A. P., Panero, M. & Terracini, B. (1973) *Il cancro in Piemonte:* 1965–69. Torino: Registro dei Tumori per il Piemonte e la Valle d'Aosta.

Armstrong, B. (1976) Recent trends in breast cancer incidence and mortality in relation to changes in possible risk factors. *Int. J. Cancer*, **17**, 204.

Beral, V. (1974) Cancer of the cervix: a sexually transmitted infection? *Lancet*, i, 1037.

Bjarnson, O., Day, N., Snaedal, G. & Tulinius, H. (1974) The effect of year of birth on the breast-cancer age-incidence curve in Iceland. *Int. J. Cancer*, **13**, 689.

Istituto Centrale di Statistica (1956–1973) *Annuario di Statistiche Demografiche*. Roma: Istat.

Istituto Centrale di Statistica (1956–1973) *Annuario di Statistiche Sanitarie*. Roma: Istat.

Istituto Centrale di Statistica (1956–1973) *Annuario Statistico Italiano*. Roma: Istat.

Istituto Centrale di Statistica (1958) *Cause di morte*, 1887–1955. Roma: Istat.

Istituto Centrale di Statistica (1976) *Sommario di Statistiche Storiche dell'Italia*, 1861–1975. Roma: Istat.

MacMahon, B., Cole, P. & Brown, J. (1973) Etiology of human breast cancer: A review. *J. Natl Cancer Inst.*, **50**, 21.

Moolgavkar, S. H., Stevens, R. G. & Lee, A. H. (1979) Effect of age on incidence of breast cancer in females. *J. Natl Cancer Inst.*, **62**, 493.

Saracci, R. & Repetto, F. (1978) Epidemiology of breast cancer. *Semin. Oncol.*, **5**, 342.

Segi, M., Noye, H. & Segi, R. (1977) *Age-adjusted death rates for cancer for selected sites (A-classification) in 43 countries in 1972*. Nagoya: Segi Inst. Cancer Epidemiol.

Stevens, R. G. & Lee, J. A. H. (1978) Tuberculosis: Generation effects and chemotherapy. *Am. J. Epidemiol.*, **107**, 120.